# Machine Learning Identifies Metabolic Dysfunction–Associated Steatotic Liver Disease in Patients With Diabetes Mellitus

**DOI:** 10.1210/clinem/dgae060

**Published:** 2024-02-08

**Authors:** Katarzyna Nabrdalik, Hanna Kwiendacz, Krzysztof Irlik, Mirela Hendel, Karolina Drożdż, Agata M Wijata, Jakub Nalepa, Oliwia Janota, Wiktoria Wójcik, Janusz Gumprecht, Gregory Y H Lip

**Affiliations:** Department of Internal Medicine, Diabetology and Nephrology, Faculty of Medical Sciences in Zabrze, Medical University of Silesia, 40-055 Katowice, Poland; Liverpool Centre for Cardiovascular Science at University of Liverpool, Liverpool John Moores University and Liverpool Heart & Chest Hospital, Liverpool L69 3BX, UK; Department of Internal Medicine, Diabetology and Nephrology, Faculty of Medical Sciences in Zabrze, Medical University of Silesia, 40-055 Katowice, Poland; Liverpool Centre for Cardiovascular Science at University of Liverpool, Liverpool John Moores University and Liverpool Heart & Chest Hospital, Liverpool L69 3BX, UK; Students’ Scientific Association by the Department of Internal Medicine, Diabetology and Nephrology, Faculty of Medical Sciences in Zabrze, Medical University of Silesia, 40-055 Katowice, Poland; Students’ Scientific Association by the Department of Internal Medicine, Diabetology and Nephrology, Faculty of Medical Sciences in Zabrze, Medical University of Silesia, 40-055 Katowice, Poland; Department of Internal Medicine, Diabetology and Nephrology, Faculty of Medical Sciences in Zabrze, Medical University of Silesia, 40-055 Katowice, Poland; Liverpool Centre for Cardiovascular Science at University of Liverpool, Liverpool John Moores University and Liverpool Heart & Chest Hospital, Liverpool L69 3BX, UK; Faculty of Biomedical Engineering, Silesian University of Technology, 41-800 Zabrze, Poland; Liverpool Centre for Cardiovascular Science at University of Liverpool, Liverpool John Moores University and Liverpool Heart & Chest Hospital, Liverpool L69 3BX, UK; Department of Algorithmics and Software, Silesian University of Technology, 44-100 Gliwice, Poland; Department of Internal Medicine, Diabetology and Nephrology, Faculty of Medical Sciences in Zabrze, Medical University of Silesia, 40-055 Katowice, Poland; Students’ Scientific Association by the Department of Internal Medicine, Diabetology and Nephrology, Faculty of Medical Sciences in Zabrze, Medical University of Silesia, 40-055 Katowice, Poland; Department of Internal Medicine, Diabetology and Nephrology, Faculty of Medical Sciences in Zabrze, Medical University of Silesia, 40-055 Katowice, Poland; Liverpool Centre for Cardiovascular Science at University of Liverpool, Liverpool John Moores University and Liverpool Heart & Chest Hospital, Liverpool L69 3BX, UK; Danish Center for Health Services Research, Department of Clinical Medicine, Aalborg University, 9220 Aalborg, Denmark

**Keywords:** diabetes, metabolic dysfunction–associated steatotic liver disease, machine learning, risk prediction

## Abstract

**Context:**

The presence of metabolic dysfunction–associated steatotic liver disease (MASLD) in patients with diabetes mellitus (DM) is associated with a high risk of cardiovascular disease, but is often underdiagnosed.

**Objective:**

To develop machine learning (ML) models for risk assessment of MASLD occurrence in patients with DM.

**Methods:**

Feature selection determined the discriminative parameters, utilized to classify DM patients as those with and without MASLD. The performance of the multiple logistic regression model was quantified by sensitivity, specificity, and percentage of correctly classified patients, and receiver operating characteristic (ROC) curve analysis. Decision curve analysis (DCA) assessed the model's net benefit for alternative treatments.

**Results:**

We studied 2000 patients with DM (mean age 58.85 ± 17.37 years; 48% women). Eight parameters: age, body mass index, type of DM, alanine aminotransferase, aspartate aminotransferase, platelet count, hyperuricaemia, and treatment with metformin were identified as discriminative. The experiments for 1735 patients show that 744/991 (75.08%) and 586/744 (78.76%) patients with/without MASLD were correctly identified (sensitivity/specificity: 0.75/0.79). The area under ROC (AUC) was 0.84 (95% CI, 0.82-0.86), while DCA showed a higher clinical utility of the model, ranging from 30% to 84% threshold probability. Results for 265 test patients confirm the model's generalizability (sensitivity/specificity: 0.80/0.74; AUC: 0.81 [95% CI, 0.76-0.87]), whereas unsupervised clustering identified high-risk patients.

**Conclusion:**

A ML approach demonstrated high performance in identifying MASLD in patients with DM. This approach may facilitate better risk stratification and cardiovascular risk prevention strategies for high-risk patients with DM at risk of MASLD.

Nonalcoholic fatty liver disease (NAFLD) is one of the most common chronic liver diseases; its prevalence has grown over recent decades and it now affects approximately 30% of adults globally (1). Because *NAFLD* does not take into account all underlying pathophysiologic conditions, Eslam et al proposed in 2020 to replace this terminology with *metabolic dysfunction–associated fatty liver disease* (MAFLD), as this highlighted the metabolic abnormalities in the condition (2). However, MAFLD still seemed to stigmatize patients with the term *fatty* and raised concerns related to mixing etiologies. Therefore, the Delphi consensus recently changed the nomenclature again and proposed *metabolic dysfunction–associated steatotic liver disease* (MASLD) (3).

One of the most important metabolic diseases among comorbidities associated with liver steatosis is type 2 diabetes mellitus (T2DM) (4). T2DM is also one of the criteria for MASLD classification. Despite the high prevalence of NAFLD (the prevalence of MASLD is not known yet since the term was introduced in June 2023) in patients with T2DM, the diagnosis is often unrecognized in clinical practice, and current risk prediction is suboptimal. The European Associations for the Study of the Liver, Diabetes and Obesity (EASL, EASD, EASO) recommended screening patients with T2DM for the presence of NAFLD because of high risk of disease progression (5) and its associations with an elevated risk of cardiovascular (CV) mortality in the long-term (6). Since CV disease (CVD) is a more common cause of death than liver disease in patients with NAFLD (7), early diagnosis is important. For the detection of liver steatosis, ultrasound is the recommended first-line diagnostic method. However, it may be difficult to encourage every patient suspected of MASLD to undergo it. The diagnostic process could be streamlined if patients are informed that new technology or refined risk stratification suggests that they are at elevated risk for liver steatosis and thus increased CV risk.

Such new technology is machine learning (ML), which may quickly analyze the most discriminative predictors differentiating patients with and without diseases, refining risk stratification (8-10). Recently, we demonstrated that a ML approach based on easy-to-obtain parameters can accurately identify patients with DM at a high risk of new CV events (11) and MAFLD patients with prevalent CVD (12). Such an approach in clinical practice can help identify patients who are at risk of MASLD, who could then be targeted for detailed liver examinations for confirmation. To the best of our knowledge, there has been no study performed to develop ML models for assessing the risk of MASLD occurrence in a well-profiled cohort of patients with DM.

## Materials and Methods

### Study Design

This is a single-center, observational study, examining patients with DM, hospitalized in the diabetology ward in Zabrze, Poland, in 2015 to 2020. This study is a part of the Silesia Diabetes-Heart Project (registered on ClinicalTrials.gov: NCT05626413), previously detailed in (11). We enrolled patients with type 1 DM (T1DM) or T2DM into the study, and exclusion criteria were terminal stages of cancer or in-hospital mortality. For this analysis, only the baseline hospitalization was considered.

### Ethical Approval

Upon admission to the hospital, all patients provided informed consent. Given that the data analyzed were sourced from an anonymized registry, further consent was not required and ethical approval was granted by the Medical University of Silesia Ethics Committee (PCN/0022/KB/126/20). The study adhered to the principles outlined in the Declaration of Helsinki. Any tests or interventions conducted during the hospitalization were part of routine medical care.

### MASLD Diagnosis

Diagnosis of MASLD was established through hepatic ultrasonography demonstrating steatosis, in conjunction with at least one of the following criteria: T2DM, a body mass index (BMI) of 25 kg/m^2^ or greater, a blood pressure reading of 130/85 mmHg or higher, pharmacological treatment for hypertension, plasma triglycerides equal to or greater than 1.7 mmol/L, or plasma high-density lipoprotein cholesterol levels lower than 1.0 mmol/L in men and 1.3 mmol/L in women or current lipid-lowering therapy (3). The ultrasonographic assessment of liver steatosis was performed using the ARIETTA 750 ultrasound system (Hitachi) with a C253 transducer. The full description of the methods is presented in Supplementary material (13).

### Predicting MASLD Using ML in Patients With DM

The process of predicting the occurrence of MASLD in patients with DM included the analysis of 80 patient parameters, including demographic characteristics (2 parameters), clinical data (DM-related [3 parameters], vascular disease-related [10], diabetic complications [3], general [3], and concomitant disease [5]), together with laboratory parameters (30) and pharmacological parameters (24) (Table 1). Prior to developing the ML methods, missing data was imputed using factorial analysis (14).

**Table 1.** Clinical patient parameters of dataset A

Parameter	Patients without MASLD (n = 744)	Patients with MASLD (n = 991)	*P* value
***Demographic parameters***			
**Age [years]**	**54.53 ± 20.61 (57.50)**	**62.48 ± 13.47 (63.00)**	**<**.**001**
Men, [n (%)]	307 (41.26%)	509 (51.36%)	<.001
***Clinical parameters***			
*Diabetes-related*			
**BMI [kg/m^2^]**	**27.30 ± 6.58 (26.01)**	**32.66 ± 6.69 (32.35)**	**<**.**001**
Duration of diabetes [years]	12.00 ± 10.13 (10.00)	10.59 ± 8.24 (10.00)	.053
**Type of diabetes [% of type 1]**	**299 (40.19%)**	**71 (7.16%)**	**<**.**001**
*Cardiovascular-related*			
Atrial fibrillation	64 (8.86%)	95 (9.87%)	.482
Carotid arteries stenosis	14 (1.88%)	15 (1.51%)	.554
Coronary artery disease	225 (30.24%)	388 (39.15%)	<.001
Heart failure	123 (16.53%)	194 (19.58%)	.104
Hypertension	443 (59.62%)	823 (83.05%)	<.001
Mean diastolic blood pressure [mmHg]	75.92 ± 7.58 (76.00)	76.59 ± 7.43 (78.00)	.018
Mean heart rate [bpm]	80.23 ± 15.42 (80.00)	80.36 ± 15.00 (80.00)	.936
Mean systolic blood pressure [mmHg]	125.87 ± 14.92 (124.00)	129.83 ± 14.74 (130.00)	<.001
Peripheral artery disease	31 (4.17%)	50 (5.05%)	.391
Stroke	64 (8.60%)	80 (8.07%)	.692
*Diabetic complications*			
Diabetic foot disease	19 (2.56%)	31 (3.13%)	.479
Diabetic peripheral neuropathy	68 (9.15%)	84 (8.48%)	.629
Retinopathy	296 (39.78%)	332 (33.50%)	.007
*General*			
Current smoker [% of yes]	119 (15.99%)	199 (20.08%)	.029
Emergency admission [% of yes]	221 (29.70%)	244 (24.65%)	.018
Number of days of hospital stay	7.05 ± 2.89 (7.00)	7.44 ± 2.73 (7.00)	.007
*Concomitant diseases*			
Chronic kidney disease	125 (16.87%)	197 (19.92%)	.103
Degenerative disease of the spine	199 (26.78%)	456 (46.01%)	<.001
Hypercholesterolemia	419 (56.32%)	694 (70.03%)	<.001
Hypertriglyceridemia	180 (24.19%)	471 (47.53%)	<.001
**Hyperuricemia**	**154 (20.70%)**	**350 (35.32%)**	**<**.**001**
***Laboratory parameters***			
**Alanine aminotransaminase [U/L]**	**23.48 ± 24.34 (18.30)**	**41.18 ± 84.07 (26.25)**	**<**.**001**
**Aspartate aminotransaminase [U/L]**	**21.28 ± 12.30 (18.50)**	**41.25 ± 110.22 (25.55)**	**<**.**001**
Basophil count [10^9^/L]	0.04 ± 0.05 (0.02)	0.04 ± 0.06 (0.03)	.003
Creatinine [mmol/L]	90.60 ± 42.38 (80.00)	94.05 ± 40.38 (82.00)	.013
CRP [mg/L]	19.01 ± 52.50 (2.51)	19.89 ± 54.09 (4.03)	<.001
eGFR [mL/min/1.73m^2^]	82.35 ± 33.10 (81.29)	77.07 ± 31.09 (76.54)	.001
Eosinophil count [10^9^/L]	0.19 ± 0.22 (0.15)	0.20 ± 0.53 (0.14)	.137
HbA1c [%]	9.10 ± 2.45 (8.66)	9.13 ± 2.33 (8.90)	.323
HCT [%]	39.45 ± 5.68 (39.90)	40.27 ± 6.06 (40.90)	<.001
Hgb [g/dL]	13.40 ± 2.09 (13.50)	13.72 ± 2.19 (14.00)	<.001
Ketones—urine sample	195 (26.97%)	160 (16.31%)	<.001
Lymphocyte count [10^9^/L]	2.11 ± 1.89 (1.94)	2.38 ± 3.77 (2.10)	.004
MCH [pg]	30.42 ± 2.84 (30.40)	30.88 ± 2.75 (30.80)	<.001
MCHC [g/dL]	33.86 ± 1.27 (33.90)	33.98 ± 1.33 (34.00)	.036
MCV [fL]	89.65 ± 6.31 (89.60)	90.85 ± 6.96 (90.50)	<.001
Mean fast. glycemia [mg/dL] first day	193.02 ± 79.69 (179.00)	197.92 ± 85.45 (179.50)	.324
Mean fast. glycemia [mg/dL] last day	133.68 ± 38.12 (129.50)	137.58 ± 34.62 (134.00)	.003
Mean post. glycemia [mg/dL] first day	173.37 ± 73.00 (163.00)	178.84 ± 60.32 (166.00)	.027
Mean post. glycemia [mg/dL] last day	138.86 ± 29.71 (136.00)	139.80 ± 30.35 (136.00)	.631
Monocyte count [10^9^/L]	0.64 ± 0.39 (0.55)	0.66 ± 0.60 (0.56)	.578
Neutrophil count [10^9^/L]	6.15 ± 3.91 (5.14)	5.79 ± 4.03 (4.91)	.028
**Platelet count [10^9^/L]**	**278.95 ± 92.69 (265.00)**	**226.94 ± 85.19 (219.00)**	**<**.**001**
Potassium [mmol/L]	4.61 ± 0.57 (4.56)	4.59 ± 0.58 (4.57)	.597
Protein—urine sample	297 (40.80%)	411 (41.73%)	.515
Red blood cell count [10^12^/L]	4.44 ± 0.79 (4.49)	4.47 ± 0.71 (4.54)	.051
Sodium [mmol/L]	138.56 ± 10.31 (139.00)	140.12 ± 41.32 (139.00)	<.001
Total cholesterol [mmol/L]	4.59 ± 1.22 (4.49)	4.64 ± 1.68 (4.43)	.388
Triglyceride [mmol/L]	1.47 ± 0.98 (1.22)	2.18 ± 2.02 (1.69)	<.001
Uric acid [mmol/L]	306.55 ± 115.03 (284.50)	343.09 ± 110.25 (329.00)	<.001
White blood cell count [10^9^/L]	9.10 ± 4.61 (8.10)	8.86 ± 5.20 (8.00)	.243
***Pharmacotherapy***			
ACEi/ARB	299 (40.19%)	612 (61.76%)	<.001
Allopurinol	123 (16.53%)	263 (26.54%)	<.001
Alpha blocker	63 (8.47%)	121 (12.21%)	.012
Amiodarone	3 (0.40%)	7 (0.71%)	.409
ASA	289 (38.84%)	562 (56.71%)	<.001
Beta blocker	315 (42.34%)	596 (60.14%)	<.001
Calcium blocker	156 (20.97%)	317 (31.99%)	<.001
Clopidogrel	35 (4.70%)	46 (4.64%)	.951
Digoxin	12 (1.61%)	16 (1.61%)	.998
DPP-4 inhibitors	111 (14.92%)	162 (16.35%)	.419
Fibrate	4 (0.54%)	28 (2.83%)	<.001
GLP-1 agonist	13 (1.75%)	22 (2.22%)	.488
Heparin	37 (4.97%)	57 (5.75%)	.478
Insulin	605 (81.32%)	761 (76.79%)	.023
PPI	203 (27.28%)	312 (31.48%)	.058
Loop diuretic	190 (25.54%)	325 (32.80%)	.001
**Metformin**	**241 (32.39%)**	**599 (60.44%)**	**<**.**001**
NOAC	40 (5.38%)	65 (6.56%)	.307
Non-loop diuretics	71 (9.54%)	202 (20.38%)	<.001
Potassium-sparing diuretics	55 (7.39%)	118 (11.91%)	.002
SGLT2 inhibitor	50 (6.72%)	153 (15.44%)	<.001
Statin	303 (40.73%)	607 (61.25%)	<.001
Sulfonylureas	195 (26.21%)	278 (28.05%)	.394
VKA	23 (3.09%)	30 (3.03%)	.939

For each parameter (if applicable), mean ± SD is reported together with the median (in parentheses). For each binary parameter, we calculated the total number of ones and the percentage of ones. The *P* values were obtained using either Mann-Whitney U-test or χ^2^ test as appropriate. The most discriminative predictors are bolded.

Abbreviations: ACEI, angiotensin-converting-enzyme inhibitor; ARB, angiotensin II receptor blocker; ASA, acetylsalicylic acid; BMI, body mass index; CRP, C-reactive protein; DPP-4, dipeptidyl peptidase 4; eGFR, estimated glomerular filtration rate; GLP-1, glucagon-like peptide-1; HbA1c, hemoglobin A1C; HCT, hematocrit; Hgb, hemoglobin; NOAC, novel oral anticoagulants; MASLD, metabolic dysfunction–associated steatotic liver disease; MCH, mean corpuscular hemoglobin; MCHC, mean corpuscular hemoglobin concentration; MCV, mean corpuscular volume; PPI, proton-pump inhibitor; SGLT-2, sodium-glucose cotransporter 2; VKA, vitamin K antagonists.

To determine the most discriminative predictors differentiating patients with and without MASLD, denoted as MASLD(+) and MASLD(-), we utilized feature selection using a χ2 test. The stability of the selected features was ensured by repeating the Monte-Carlo simulation 1000 times: in each experiment, we randomly selected 80% of all patients from the MASLD(+) and MASLD(-) groups, for whom the most discriminative features were selected. For those predictors, the *P* value obtained by a χ2 test was less than 0.05, and the final set of features included the predictors which were selected as discriminative in all 1000 independent runs.

The most discriminative parameters were used to predict the occurrence of MASLD by a multiple logistic regression (MLR) model, and the cutoff value was extracted from the receiver operating characteristic curve (ROC) using the Index of Union technique (15). Unsupervised hierarchical clustering was performed for all patients using the most discriminative predictors. The optimal number of groups was determined using the Calinski-Harabasz criterion (16).

To evaluate the performance of the algorithms, we use sensitivity, specificity, and the percentage of correctly classified (CC) patients with and without MASLD. For the MLR model, the ROC curve was determined and the area under this curve (AUC) was calculated, together with its 95% CI. The clinical usefulness of the proposed model was assessed using decision curve analysis (DCA). The MLR models were also validated using an independent test set including patients who were never used at any stage of developing the ML model. Clusters were investigated by comparing the parameter values in the obtained groups, as well as the percentage of MASLD(+) patients in each cluster. Statistical analysis, feature selection and visualizations were carried out in MATLAB R2023a.

## Results

**Figure 1.**
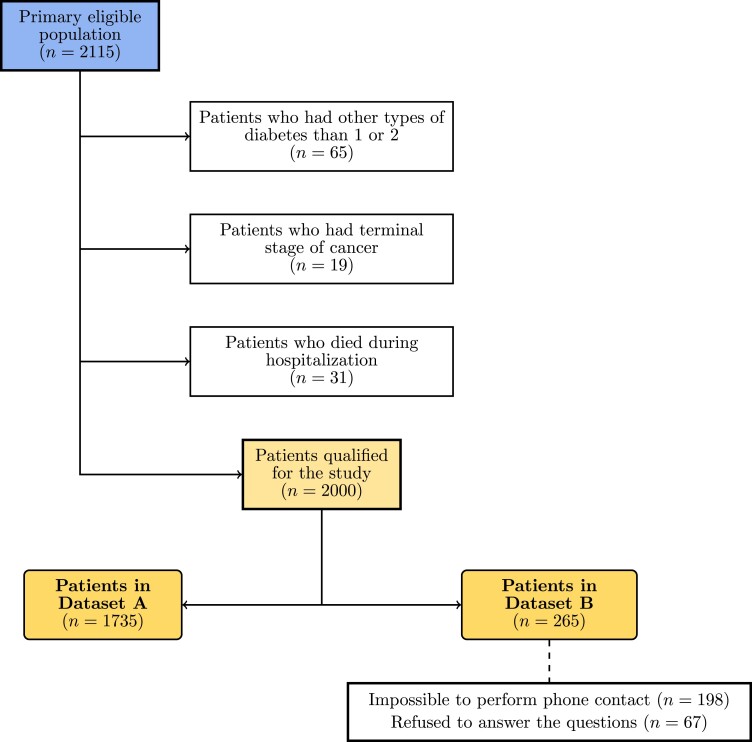
Patient flowchart.

Out of 2115 eligible patients with DM, we included 2000 patients in this study (mean age, 58.85 ± SD 17.37 years; 48% women) (Fig. 1). There were 1735 patients who served for feature selection and for training the models using the most discriminating features (Dataset A) (Table 1). A subset of 265 patients (Dataset B) (Supplementary Table S2) was used as a test set to verify the generalizability of the models (13). Among Dataset A patients (n = 1735), there were 370 (21%) patients with T1DM and 1383 with T2DM; 991 (57%) patients were diagnosed with MASLD. Dataset B (n = 265) consisted of 58 (22%) patients with T1DM and 207 (78%) with T2DM. Here, MASLD was diagnosed in 137 (52%) patients.

### Feature Selection

Feature selection resulted in 8 most discriminative predictors—their values significantly differ across the MASLD(+) and MASLD(-) groups (Table 1 and Supplementary Fig. S1): age, BMI, type of DM, alanine aminotransaminase (ALT), aspartate aminotransaminase (AST), platelet count, treatment with metformin, and hyperuricemia (13). The performance of the MLR model for identifying MASLD(+) and MASLD(-) patients is presented in Table 2.

**Table 2.** The results of predicting MASLD in patients with diabetes based on 8 most discriminative and all (80) features using the MLR model

Subset	Method	Sensitivity	Specificity	CC with MASLD [%]	CC without MASLD [%]	CC all [%]
**Dataset A** **(n = 1735)**	**MLR (8 features)**	0.75	0.79	75.08%	78.76%	76.66%
	**MLR (80 features)**	0.82	0.75	82.24%	75.00%	79.14%
**Dataset B** **(n = 265)**	**MLR (8 features)**	0.80	0.74	79.56%	74.56%	74.22%
	**MLR (80 features)**	0.77	0.74	77.37%	74.23%	74.21%

Abbreviations: CC, correctly classified; MASLD, metabolic dysfunction–associated steatotic liver disease; MLR, multiple logistic regression.

### Evaluating the Multiple Logistic Regression ML Model

**Figure 2.**
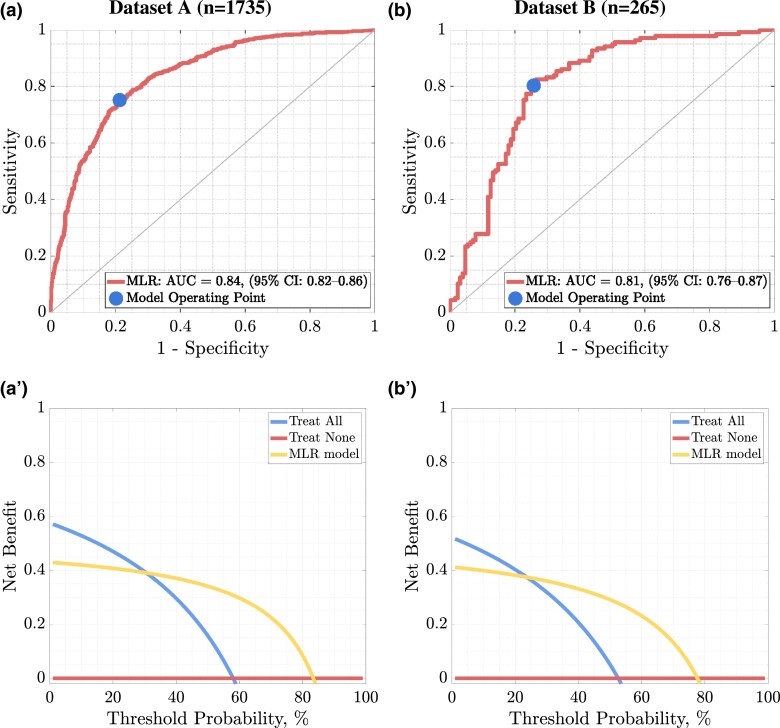
The ROC curve (a) together with the DCA (a′) determined for the results of the MLR model fitted to the 8 selected features for dataset a, while the ROC (b) and the DCA (b′) curves were obtained for dataset b. The 45° curve through the origin in the case of the ROC curve shows the discriminant ability of the classifier no better than random selection.

Of the cohort, 744/991 (75.08%) MASLD(+) and 586/744 (78.76%) MASLD(-) patients were correctly identified, which amounts to 1330/1735 (76.66%) of the correct predictions among all Dataset A patients (hence, for 1330/1735 patients, their MASLD status was correctly identified using a ML model). The high performance of the MLR model is also reflected in its sensitivity (0.75) and specificity (0.79). The classifier was evaluated using ROC (Fig. 2a and 2a′) and DCA (Fig. 2b and 2b′) curves. AUC was 0.84 (95% CI, 0.82-0.86), while the clinical utility based on DCA shows a higher utility of the model in terms of net benefit for alternative strategies (treating none and all patients), ranging from 30% to 84% threshold probability.

**Figure 3.**
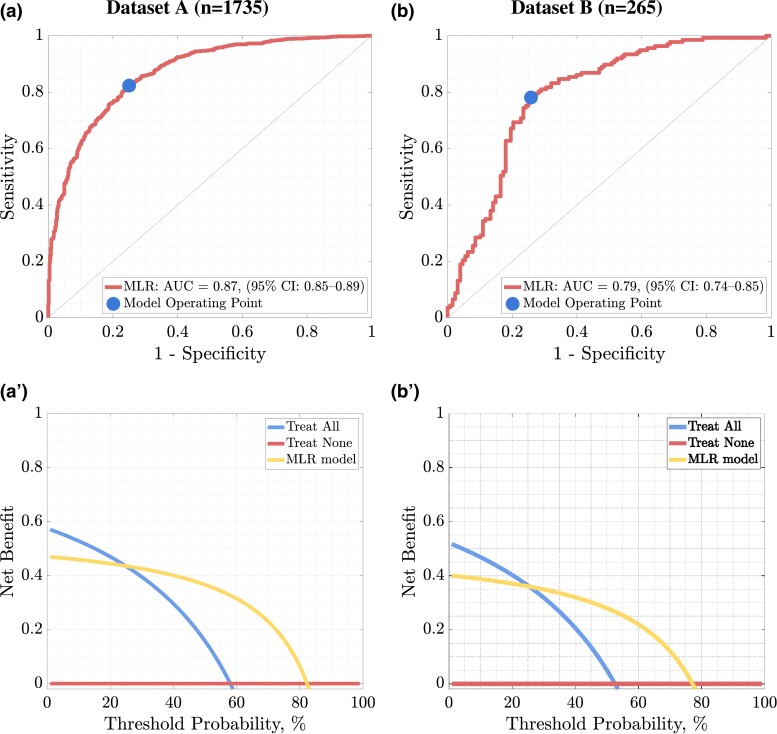
The ROC curve (a) together with the DCA (a′) determined for the results of the MLR model fitted to all (80) features for dataset a, while the ROC (b) and the DCA (b′) curves were obtained for dataset b. The 45° curve through the origin in the case of the ROC curve shows the discriminant ability of the classifier no better than random selection.

Utilizing all 80 predictors led to AUC of 0.87 (95% CI, 0.86-0.89), with 815/991 (82.24%) and 586/744 (75.00%) correctly identified MASLD(+) and MASLD(-) patients; thus, 1373/1735, 79.14% of all patients were appropriately classified as those with or without MASLD (Table 2). In Dataset B, we obtained an AUC of 0.81 (95% CI, 0.76-0.87) for MLR operating on the 8 most discriminative features, and AUC of 0.79 (95% CI, 0.74-0.85) for the model exploiting all available predictors; this shows the importance of feature selection (Fig. 3). DCA shows a higher utility of the ML model in terms of net benefit for alternative treatment strategies, ranging from 24% to 78% threshold probability for Dataset B.

### Unsupervised Hierarchical Clustering

The patients of both datasets were subjected to hierarchical clustering based on the 8 most discriminative features, with the optimal number of 10 clusters. The distribution of those parameters for patients included in all clusters are in Table 3 and Supplementary Fig. S3 for Dataset A, and in Supplementary Table S2 and Supplementary Fig. S4 for Dataset B (13). This showed that there are clusters corresponding to higher-risk MASLD patients which are consistently elaborated for both sets. Such profiling of patients, based on 8 easy-to-obtain parameters, may help better design the treatment pathway to those patients who are at a higher MASLD risk.

**Table 3.** **The results of hierarchical patient (dataset A) clustering based on 8 most discriminative predictors (the clusters are indicated as C1-C10)**.

Parameter	C1 (n = 303)	C2 (n = 400)	C3 (n = 1)	C4 (n = 44)	C5 (n = 153)	C6 (n = 618)	C7 (n = 13)	C8 (n = 201)	C9 (n = 1)	C10 (n = 1)	*P* value
Age [years]	52.99 ± 18.34 (54.00)	57.36 ± 18.43 (61.00)	67.00 ± 0.00 (67.00)	56.09 ± 18.72 (55.00)	64.13 ± 15.32 (67.00)	62.52 ± 15.33 (64.00)	70.62 ± 10.87 (72.00)	57.03 ± 17.41 (59.00)	44.00 ± 0.00 (44.00)	81.00 ± 0.00 (81.00)	<.001
Alanine aminotransaminase [U/L]	20.83 ± 9.24 (19.30)	29.11 ± 20.27 (22.80)	909.00 ± 0.00 (909.00)	180.48 ± 85.08 (164.00)	30.56 ± 18.73 (25.40)	29.44 ± 18.39 (24.05)	27.72 ± 18.95 (23.90)	29.12 ± 22.69 (21.70)	546.80 ± 0.00 (546.80)	2170.00 ± 0.00 (2170.00)	<.001
Aspartate aminotransaminase [U/L]	20.97 ± 8.32 (19.40)	26.83 ± 16.62 (22.05)	241.90 ± 0.00 (241.90)	154.29 ± 82.17 (134.05)	37.81 ± 26.48 (28.40)	27.19 ± 14.35 (22.65)	26.21 ± 20.36 (18.10)	30.03 ± 23.44 (22.80)	1340.00 ± 0.00 (1340.00)	3023.00 ± 0.00 (3023.00)	<.001
BMI [kg/m^2^]	28.87 ± 6.66 (27.90)	30.33 ± 7.04 (29.82)	25.28 ± 0.00 (25.28)	29.57 ± 5.87 (29.48)	30.42 ± 6.39 (30.43)	31.32 ± 6.75 (31.01)	27.25 ± 4.98 (27.7)	28.98 ± 7.22 (28.53)	25.60 ± 0.00 (25.60)	23.96 ± 0.00 (23.96)	<.001
Hyperuricemia	59 (19.47%)	126 (31.50%)	0 (0.00%)	10 (22.73%)	56 (36.60%)	194 (31.39%)	3 (23.08%)	55 (27.36%)	0 (0.00%)	1 (100.00%)	.003
Metformin	129 (42.57%)	193 (48.25%)	0 (0.00%)	5 (11.36%)	64 (41.83%)	362 (58.58%)	6 (46.15%)	81 (40.30%)	0 (0.00%)	0 (0.00%)	<.001
Platelet count [10^9^/L]	249.61 ± 16.99 (252.00)	298.84 ± 22.40 (299.00)	156.00 ± 0.00 (156.00)	189.30 ± 81.59 (186.50)	115.01 ± 35.81 (128.00)	197.76 ± 24.49 (197.00)	716.54 ± 142.54 (683.00)	395.65 ± 53.68 (385.00)	66.00 ± 0.00 (66.00)	113.00 ± 0.00 (113.00)	<.001
Type of diabetes [% of type 1]	119 (39.27%)	100 (25.00%)	0 (0.00%)	3 (6.82%)	13 (8.50%)	85 (13.75%)	1 (7.69%)	48 (23.88%)	1 (100.00%)	0 (0.00%)	<.001
**MASLD**	**117 (38.61%)**	**188 (47.00%)**	**1 (100.00%)**	**40 (90.91%)**	**140 (91.50%)**	**416 (67.31%)**	**3 (23.08%)**	**84 (41.79%)**	**1 (100.00%)**	**1 (100.00%)**	**<**.**001**

For each parameter (if applicable), mean ± SD is reported together with the median (in parentheses). For each binary parameter, we calculated the total number of ones and the percentage of ones. The *P* values were obtained using the Kruskal-Wallis test with post hoc Dunn. The row with the target (MASLD) is boldfaced.

## Discussion

The key findings of our investigation are as follows: (i) we determine 8 (out of 80) most discriminative patient parameters (age, BMI, type of DM, ALT, AST, hyperuricemia, platelet count, and metformin treatment) which enabled to identify patients who are most likely to present with MASLD using a MLR model; and (ii) on the basis of 8 parameters, we clustered individuals with similar phenotypes in order to stratify risk of MASLD presence.

The potential clinical utility of our approach lies in the independent role of MASLD as predictor of CVD, which is distinct and additive to the risks associated with T2DM (17). The successful identification of MASLD in DM patients using our ML model indicates an elevated CVD risk, surpassing the inherent risks of DM. Hence, accurate identification of MASLD enables more nuanced CVD risk stratification, essential for tailoring preventive and therapeutic strategies in DM patients. This becomes particularly actionable for patients with DM with the availability of SGLT2 inhibitors and GLP-1 receptor agonists, proven to reduce CVD risk (18).

Since MASLD is only recently established nomenclature, we will refer to studies examining NAFLD/MAFLD given that those studying MASLD are not available yet. All of the parameters that were determined in our study are easy to obtain and interpret in clinical practice and mainly related to the metabolic aspect of MASLD. Age was one of the parameters determined in feature selection implicating that the older age is related to a higher MASLD risk. Data from the studies performed recently are not uniform in this term, where the incidence rate of NAFLD assessed annually was higher in people at least 50 years of age (5.5%) compared with those younger than 50 years (3.5%) (19), and varied across age groups in Chinese (20) and Israeli population (21). It can therefore be assumed that the NAFLD incidence rate may be race-related and would differ by age; however, the data related to MASLD are not available yet.

Excessive body weight is a factor included in the definition of MASLD. In the present analysis, BMI was identified as an important parameter, where higher BMI indicated the elevated risk of MASLD. This confirms what is already known for NAFLD, as an increase in BMI was a risk factor for new onset of NAFLD (19), and that the risk increases with an increasing BMI (22). Moreover, our model pinpoints the type of diabetes as a relevant parameter, specifically highlighting associations between T2DM and MASLD risk. Our analysis also brings attention to T1DM, a condition where NAFLD is much less prevalent (23) but is especially important in relation to recent data revealing that the 10-year estimated CVD risk is much higher in T1DM patients with hepatic steatosis and significant fibrosis when compared to those with hepatic steatosis alone or without steatosis (24). Moreover, prior cross-sectional studies indicate an association between NAFLD and an increased risk of CVD in T1DM, even after adjusting for traditional risk factors (25, 26). Furthermore, in patients with T1DM, NAFLD has been linked to chronic kidney disease (27), and a connection between MASLD and retinopathy has been established (28). This suggests that the ability to diagnose MASLD in T1DM could facilitate more comprehensive or frequent screening for these complications in this high-risk population.

Other parameters determined as those identifying patients with MASLD were higher values of ALT and AST. This is important, given that incorporating liver enzymes activity into assessment of patients with NAFLD can help categorize them as low or high risk for advanced fibrosis stage and metabolic-associated comorbidities (29). Also, a lower platelet count was another factor related to the presence of MASLD. This has a pathophysiological basis as platelet synthesis is regulated by thrombopoietin, a glycoprotein hormone synthesized in the liver and an inverse correlation has been reported between the degree of hepatitis and the platelet count (30). Indeed, thrombocytopenia has been related to NAFLD (31) and platelet counts could even serve as biomarker of the severity of fibrosis in NAFLD patients (32).

The most important factor associated with prognosis in patients with NAFLD is liver fibrosis (33). The gold standard of fibrosis diagnosis remains liver biopsy (34), but it is an invasive procedure so difficult to implement in all patients with NAFLD. Thus, there has been a clinical scoring tool developed, that is, the fibrosis-4 index, and recommended for noninvasive detection of advanced liver fibrosis (29, 35). In the light of presented outcomes, our model identified patients based on all these parameters and more, that constituted the screening tool for advanced fibrosis. While this is a notable observation, it does not imply that the model possesses the capability to assess fibrosis risk per se, which needs elastography/liver biopsy. Another important metabolic abnormality is hyperuricemia which we identified as a parameter determining MASLD. It confirms prior observations that elevated uric acid was a risk factor for NAFLD (36) and an independent predictor for NAFLD (37).

Our feature selection discriminated also a parameter related to DM pharmacotherapy where high-risk patients for MASLD were those who were treated with metformin. This association seems surprising because metformin has been shown to have a positive impact on liver function in NAFLD (38). On the other hand, other studies analyzing long-term metformin therapy did not show any protective effects on the liver histology (39) or a moderate one (40). Our results also confirm a real-world study, where metformin treatment caused a higher risk of developing NAFLD (41).

Finally, our predictive model based on 8 parameters correctly classified over 76% of patients, indicating its substantial capability in identifying MASLD. Beyond this metric, the model's discriminative power is further evidenced by an AUC of 0.84. The significance of this model extends into its clinical utility, as demonstrated by DCA, which reveals the model's applicability across a wide range of threshold probabilities (30%-84%). This indicates the model's relevance in varying clinical contexts, enabling healthcare professionals to apply it effectively across different patient risk profiles. For instance, in a clinical context where early detection is prioritized and diagnostic tests available, a lower threshold probability might be chosen, making the model sensitive to even moderate risks of MASLD.

When all 80 parameters were utilized by the model, the accuracy of identifying patients with MASLD were higher, but such large number of parameters is difficult to obtain in clinical settings, and the model is more likely to overfit (due to the ratio of the number of features to the number of observations) and unable to generalize. The MLR model was verified using an independent test set of 265 patients, for which the obtained metrics confirm the generalization capabilities of the model operating on 8 patients’ parameters. The clinical utility determined using DCA further highlights the clinical usefulness of the ML model.

Apart from determining the features indicating the risk for MASLD patients, our cluster analysis performed for 1735 patients from Dataset A divided them into 10 clusters. Of these clusters, C3, C9, and C10 containing a single patient appeared to be of the highest risk for MASLD and differed significantly in the analyzed parameters compared to the rest of the cohort. Such significantly different clusters were similarly visible in the test set containing patients from Dataset B, where the number of clusters was kept consistent with the optimal number of clusters determined for Dataset A (ie, 10). Albeit the differences in patient parameters are not necessarily statistically significant, qualitative analysis reveals that 3 clusters (C3, C9, and C10) contained patients with visibly different clinical characteristics than the other clusters. These differences in parameters in those 3 clusters are related to elevated activity of liver enzymes and low platelet count which are the parameters inseparable of the liver metabolic function and are used in fibrosis-4 index for assessing the fibrosis risk (42).

Our unsupervised ML distinguished the cluster of patients at a high risk of MASLD based on these parameters. This analysis highlights the importance of unsupervised hierarchical clustering in profiling patients with DM, and the possibility of extracting patient group clusters with discriminative clinical parameters. Moreover, other clusters indicate patients who are not at high risk of MASLD (clusters C1, C2, C7, and C8), where the risk of having MASLD was lower than 50%. For the 265 patients from Dataset B, clustering resulted in similarly consistent groups, indicating the patients who are at a higher risk of MASLD.

### Limitations

Although the results obtained over the set of 265 test patients confirm the generalizability of the models, this was a single-center study, hence validating the models over a larger and more general cohort could robustify our findings. Moreover, neither elastography nor liver biopsy was performed; therefore, there is no information about the stage of MASLD. Ultrasonography offers the qualitative rather than quantitative assessment of liver fat content and cannot assess fibrosis, limiting the understanding of the disease's severity. We acknowledge that the lack of waist circumference and high-density lipoprotein cholesterol measurements might lead to a slight underestimation of MASLD. However, the majority of enrolled patients are at least overweight or have T2DM which classifies them for MASLD diagnosis if other criteria are fulfilled.

## Conclusion

A ML approach demonstrated high performance in identifying MASLD in patients with DM. This approach may facilitate better risk stratification and cardiovascular risk prevention strategies for high-risk patients with DM at risk of MASLD.

## Data Availability

Some or all datasets generated during and/or analyzed during the current study are not publicly available but are available from the corresponding author on reasonable request.

## Abbreviations

ALT: alanine aminotransferase
AUC: area under the curve
AST: aspartate aminotransferase
BMI: body mass index
CV: cardiovascular
CVD: cardiovascular disease
DCA: decision curve analysis
DM: diabetes mellitus
MAFLD: metabolic-associated fatty liver disease
MASLD: metabolic dysfunction–associated steatotic liver disease
ML: machine learning
MLR: multiple logistic regression
NAFLD: nonalcoholic fatty liver disease
ROC: receiver operating characteristic
T1DM: type 1 diabetes mellitus
T2DM: type 2 diabetes mellitus
